# Artificial miRNA inhibition of phosphoenolpyruvate carboxylase increases fatty acid production in a green microalga *Chlamydomonas reinhardtii*

**DOI:** 10.1186/s13068-017-0779-z

**Published:** 2017-04-13

**Authors:** Chaogang Wang, Xi Chen, Hui Li, Jiangxin Wang, Zhangli Hu

**Affiliations:** 1 Guangdong Technology Research Center for Marine Algal Bioengineering, College of Life Sciences, Shenzhen University, Shenzhen, 518060 People’s Republic of China; 2 Guangdong Key Laboratory of Plant Epigenetics, College of Life Sciences, Shenzhen University, Shenzhen, 518060 People’s Republic of China; 3 Nanshan District Key Lab for Biopolymers and Safety Evaluation, Shenzhen University, Shenzhen, 518060 People’s Republic of China

**Keywords:** *Chlamydomonas reinhardtii*, Lipids, Biodiesel, amiRNA, *CrPEPC1*, *CrPEPC2*

## Abstract

**Background:**

Nutrient limitation, such as nitrogen depletion, is the most widely used method for improving microalgae fatty acid production; however, these harsh conditions also inhibit algal growth significantly and even kill cells at all. To avoid these problems, we used artificial microRNA (amiRNA) technology as a useful tool to manipulate metabolic pathways to increase fatty acid contents effectively in the green microalga *Chlamydomonas reinhardtii*. We down-regulated the expression of phosphoenolpyruvate carboxylase (PEPC), which catalyzes the formation of oxaloacetate from phosphoenolpyruvate and regulates carbon flux.

**Results:**

amiRNAs against two *CrPEPC* genes were designed and transformed into *Chlamydomonas* cells and amiRNAs were induced by heat shock treatment. The transcription levels of amiRNAs increased 16–28 times, resulting in the remarkable decreases of the expression of *CrPEPCs*. In the end, inhibiting the expression of the *CrPEPC* genes dramatically increased the total fatty acid content in the transgenic algae by 28.7–48.6%, which mostly increased the content of C16–C22 fatty acids. Furthermore, the highest content was that of C18:3n3 with an average increase of 35.75%, while C20–C22 fatty acid content significantly increased by 85–160%.

**Conclusions:**

Overall our results suggest that heat shock treatment induced the expression of amiRNAs, which can effectively down-regulate the expression of *CrPEPCs* in *C. reinhardtii*, resulting in an increase of fatty acid synthesis with the most significant increase occurring for C16 to C22 fatty acids.

**Electronic supplementary material:**

The online version of this article (doi:10.1186/s13068-017-0779-z) contains supplementary material, which is available to authorized users.

## Background

Biodiesel has become the focus of the world’s new energy research to replace petroleum diesel as it comes from a wide variety of sources, is biodegradable [1], has a high calorific value, and above all is environmentally friendly [2, 3]. Microalgae are the biodiesel source of choice with high growth rate, high oil production, and ease of cultivation [4, 5], which make them the most promising biodiesel material [6]. However, the lipid content in microalgae must be improved if biodiesel from microalgae is to be used for commercial scale production in the future [7, 8].

**Fig. 1:**
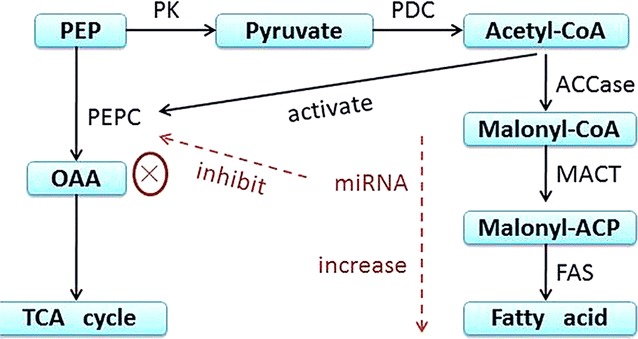
Inhibition of PEPC by miRNA promotes the synthesis of fatty acids

In recent years, increasing lipid content in microalgae through genetic engineering techniques has been tried. However, the expression of acetyl coenzyme A carboxylase (ACCase) and fatty acid synthase (FAS) from higher plants in microalgae did not achieve the aim of dramatically increasing the oil content [9, 10]. Meanwhile, previous studies showed that there was a negative correlation between the activities of phosphoenolpyruvate carboxylase (PEPC), a key enzyme of the amino acid metabolic pathway, and lipid accumulation because of sharing its ACCase with a common substrate, pyruvate [11, 12]. Therefore, it is safe to propose that the inhibition of the activity of PEPC may promote the flow of carbon to fatty acid synthesis, thereby enhancing the oil content of cells (Fig. 1).

Among numerous gene-silencing systems, microRNAs (miRNAs) are receiving much attention because of their high efficiency and accuracy. The miRNAs guide post-transcriptional regulation by means of targeted RNA degradation and translational arrest [13, 14]. Hence, amiRNA (artificial microRNA)-mediated knockdown technology as a novel technology has opened up the possibility of specifically down-regulating a gene of interest, avoiding problems associated with silencing of off-targets by siRNAs [15, 16]. *Chlamydomonas reinhardtii*, a unicellular green alga from the phylum *Chlorophyta*, is an ideal model system for studying the role of miRNAs in regulating gene expression because it has well-established genetic engineering systems in nucleus, chloroplast, and mitochondria [17, 18]. For example, pyruvate formate lyase (PFL1) expression was reduced by 80–90% at the transcriptional level by a designed amiRNA in *Chlamydomonas* [19], indicating that the technique amiRNA broadens the application prospects in the field of gene regulation in microalgae.

It is well known that there were two subtypes of *CrPEPC* enzymes, named as *CrPEPC1* and *CrPEPC2*, in *C. reinhardtii* [20, 21]. Here we designed amiRNAs, based on *CrPEPC1/2* sequences, to reduce their expression and explored their potential for increasing fatty acid accumulation in *C. reinhardtii*. This is the first report on the use of novel amiRNAs to silence *CrPEPCs* and improve the accumulation of lipid in *C. reinhardtii*. The transcription levels of amiRNA-PEPC1 and amiRNA-PEPC2 were drastically increased when induced by heat shock (HS) and the transcription levels of *CrPEPC1* and *CrPEPC2* were reduced accordingly, leading to an increase in fatty acid content.

## Results

### Screening and identification of transgenic mutants

The recombinant vectors pH-aRP1 and pH-aRP2 were introduced into the cell wall-deficient *C. reinhardtii* strain CC-849 by a “glass-bead” method, using zeomycin (10 μg/mL) as the selective antibiotic. The positive transformants were then verified by genomic DNA-PCR amplification and Sanger sequencing (see Additional file 1: Figure S1). Individual transformants were verified and selected for further study. Mutant strains with the names such as aRP1.1 and aRP1.2 were derived from individual amicroRNA-PEPC1 transformants, and aRP2.1, aRP2.2, and aRP2.3 were from amicroRNA-PEPC2 transformants. The growths of WT and transgenic algae were similar and the highest biomass was around 8 × 10^6^ cells/mL for all strains, indicating that the transformed genes had no effect on the growth of transgenic algal cells (see Additional file 2: Figure S2).

### Effect of heat shock (HS) on amiRNA transcription in transgenic algae

**Fig. 2:**
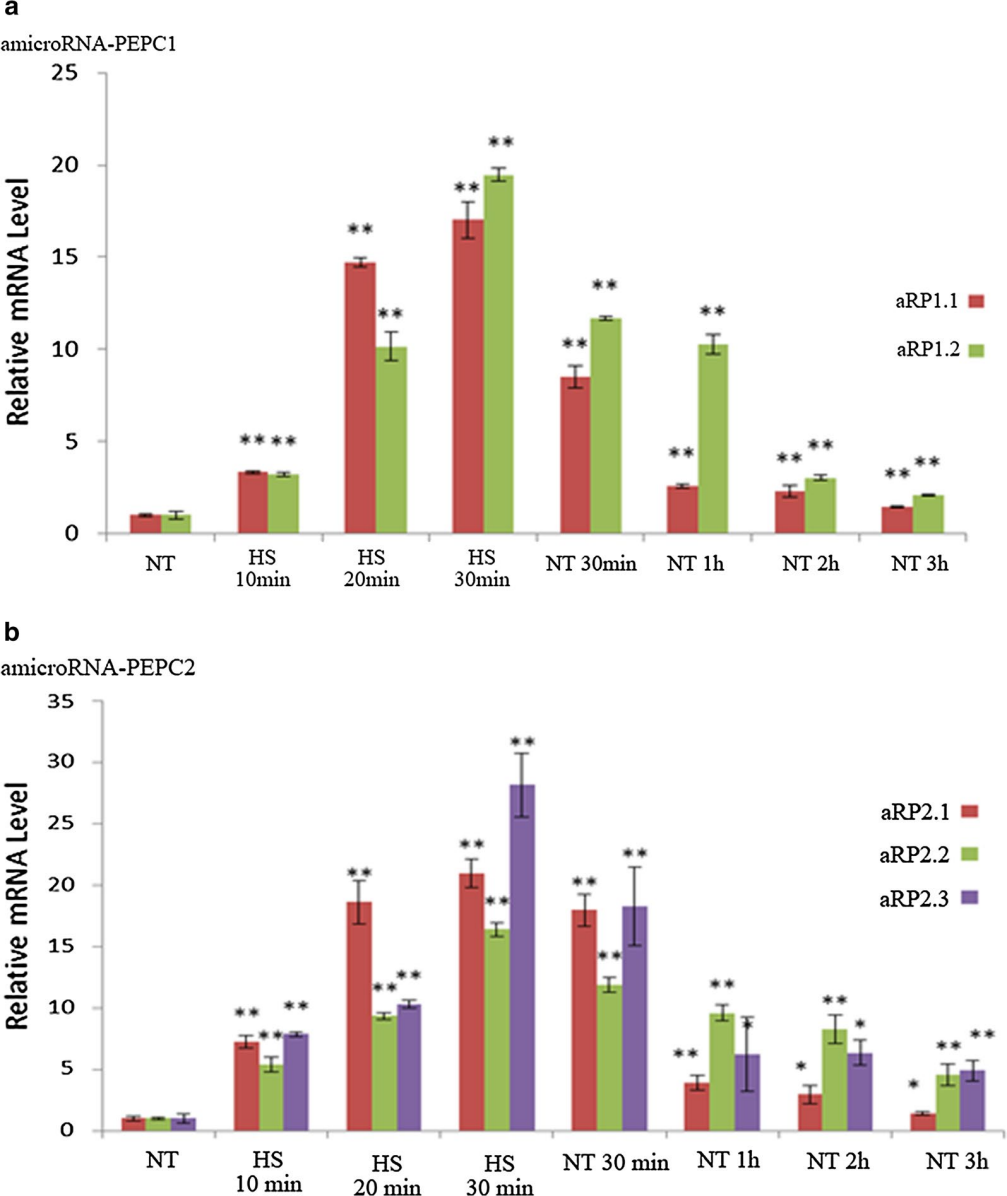
Effects of heat shock on amicroRNA-PEPC1/2 expression levels. *NT* indicates a normal condition; *HS* indicates heat shock; aRP1.1 and aRP1.2 represent two different strains of amicroRNA-PEPC1 (**a**); aRP2.1, aRP2.2, and aRP2.3 represent three different strains of amicroRNA-PEPC2 (**b**) transformants; *asterisk* and *double asterisk* indicate significantly different amicroRNA-PEPC1/2 gene expression in transgenic algae when compared with NT

Since the amiRNA vector contains an Hsp70A heat shock-responsive promoter, amiRNA expression level after HS treatment was evaluated using quantitative RT-PCR, with U4 snoRNA as the internal reference. The 30-min HS increased the amicroRNA-PEPC1 (aRP1) transcription level to 17–19 times that of the control (Fig. 2a). When the algae were cooled down to normal conditions (25 °C), the aRP1 expression level declined, but still remained three times higher than the control. Similarly, heat shock also induced amicroRNA-PEPC2 (aRP2) expression, at 16–28 times higher than the control (Fig. 2b).

### Effects of HS on *CrPEPC* genes’ expression in transgenic algae

**Fig. 3:**
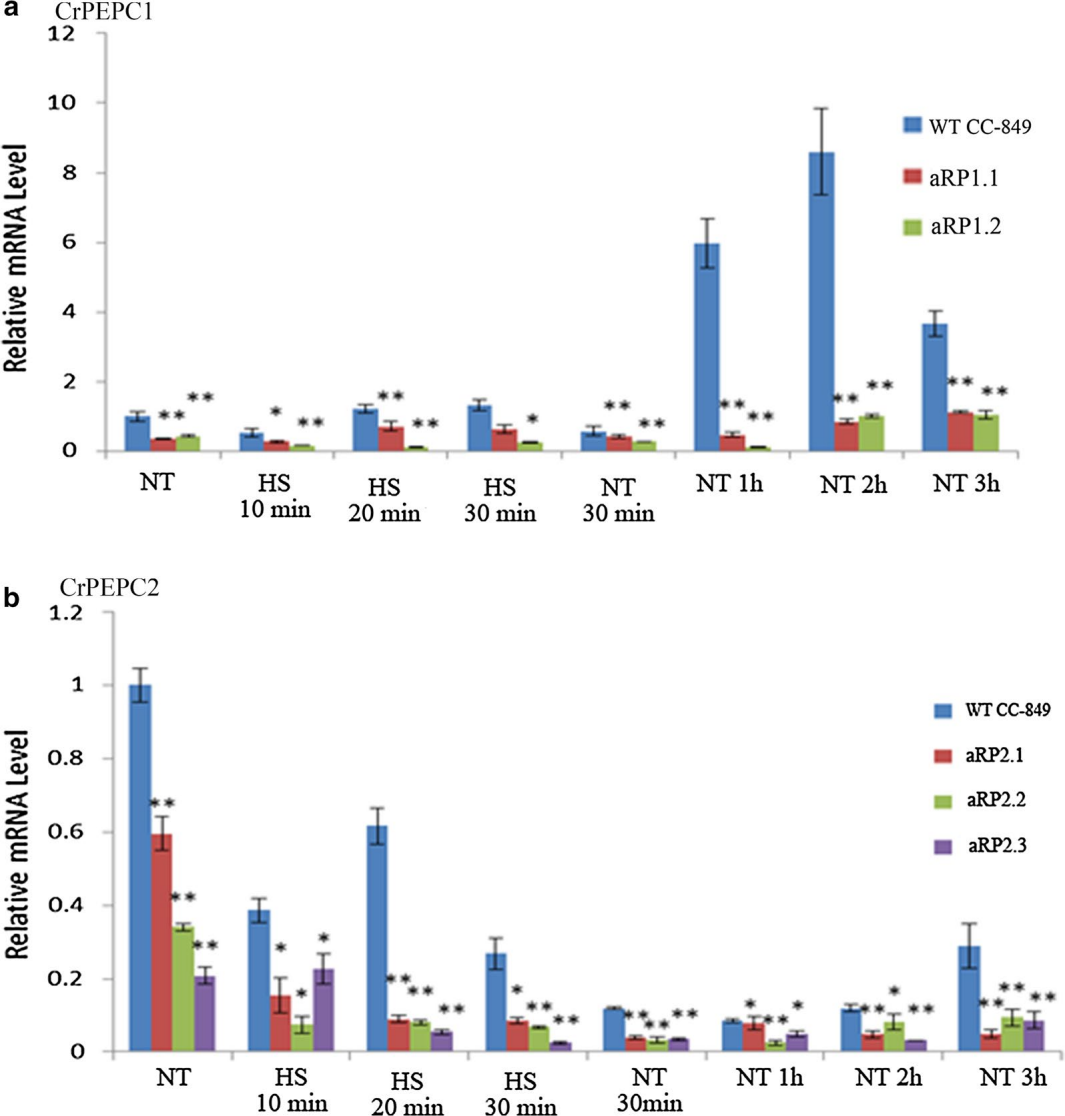
Heat shock-reduced transcription level of *CrPEPC1/2.* **a** aRP1.1 and aRP1.2 represent two different strains of amicroRNA-PEPC1 transformant; **b** aRP2.1, aRP2.2, and aRP2.3 represent three different strains of amicroRNA-PEPC2 transformant; *NT* indicates normal condition; *HS* indicates heat shock; *asterisk* and *double asterisk* indicate CrPEPC genes’ expression in transgenic algae compared to that in non-transgenic *C. reinhardtii* CC-849 on the gene expression level significantly

After HS, the transcription level of *CrPEPC1* was increased quickly in the WT, and after 2 h it reached the highest level when cooled down to the normal conditions (Fig. 3a). In contrast, in *Chlamydomonas* mutants with amicroRNA-PEPC1 (aRP1.1–aRP1.2), *CrPEPC1* transcript levels were dramatically reduced to only 7–9.8% of the control after HS treatment and returned to normal conditions (Fig. 3a). These results indicated that aRP1 transformation in *Chlamydomonas* inhibits the expression of the target gene *CrPEPC1*.

Interestingly, in the WT algae, *CrPEPC2* expression under HS was strikingly different from *CrPEPC1* expression, suggesting different HS-triggered regulation of *CrPEPC1* and *CrPEPC2* in *Chlamydomonas*. Similar to *CrPEPC1* in transgenic aRP2 cells, *CrPEPC2* gene expression levels under the same conditions were reduced to less than that of the control group. For instance, under normal conditions the *CrPEPC2* expression level decreased by 80% in aRP2 strain compared to the WT. In addition, 30 min after the HS the *CrPEPC2* expression level in transformant aRP2.3 was reduced by 90% compared to the control, indicating that the amiRNA aRP2 significantly reduced *CrPEPC2* transcripts in *Chlamydomonas*. The HS treatment reduced *CrPEPC2* in both WT and transgenic cells. After return to normal conditions, *CrPEPC2* gene expression in transgenic algae aRP2.1, aRP2.2, and aRP2.3 was reduced to 30–70% that of the control group. Based on the data analysis, it was confirmed that *CrPEPC2* gene expression was suppressed by overexpressed amiRNAs in the aRP2 strains (Fig. 3b).

### Fatty acid composition increases in genetically modified algae after HS treatment

Fatty acids (FAs) were extracted from WT and all transgenic algal cells under normal conditions and after HS. In WT cells under routine culture condition, fatty acid composition analysis indicated that the major long-chain fatty acids C16:0, C16:4, and C18:3n3 are the most abundant in proportion to the total fatty acid (TFA), accounting for up to 80.97%. Under normal conditions, the TFA contents of genetically modified algae strains including aRP1 and aRP2 strains normally increase by about 3–23% (see Table 1 for more details). TFA were further increased after HS treatments. More HS treatments were carried out and more TFA were produced in algal cells. For example, the TFA content in WT was 85.5 ± 1.5, 93.8 ± 3.01, and 106.8 ± 12.2 mg/g (dry weight, DW), after zero, one, and three heat shocks, respectively (see Tables 1, 2, 3 for more details).

**Table 1:** Fatty acids and TFA contents (% cell dry weight) in transgenic strains with amicroRNA-PEPCs as analyzed by GC–MS

Fatty acids	CC-849	aRP1.1	aRP1.2	aRP1.3	aRP1.4	aRP2.1	aRP2.2	aRP2.3
C12:0	0.046 ± 0.001	0.053 ± 0.001	0.059 ± 0.001	0.165 ± 0.029	0.046 ± 0.003	0.00	0.00	0.035 ± 0.031
C14:0	0.234 ± 0.004	0.29 ± 0.012	0.293 ± 0.022	0.369 ± 0.021	0.233 ± 0.032	0.254 ± 0.015	0.246 ± 0.022	0.314 ± 0.055
C16:0	15.46 ± 0.477	16.411 ± 0.685	18.063 ± 0.277^a^	18.602 ± 1.161^a^	15.054 ± 0.19	21.709 ± 1.005^a^	21.268 ± 1.344^a^	19.14 ± 1.667^a^
C16:1	2.247 ± 0.033	3.162 ± 0.117^b^	3.231 ± 0.465	3.849 ± 0.377^a^	2.36 ± 0.212	2.571 ± 0.164	2.838 ± 0.117^a^	2.727 ± 0.32
C18:0	2.12 ± 0.066	2.561 ± 0.085^a^	2.22 ± 0.194	2.539 ± 0.18^a^	2.08 ± 0.205	2.544 ± 0.165^a^	2.115 ± 0.247	2.232 ± 0.286
C16:4	22.987 ± 0.097	25.535 ± 0.862^a^	25.67 ± 1.452^a^	25.852 ± 1.363^a^	24.25 ± 1.418	25.057 ± 1.338	27.57 ± 0.696^b^	28.82 ± 1.088^b^
C18:1 t	0.818 ± 0.041	1.233 ± 0.108^b^	1.769 ± 0.414	2.649 ± 0.633^a^	0.91 ± 0.089	1.283 ± 0.109^b^	1.235 ± 0.295	0.969 ± 0.165
C18:2 t	3.353 ± 0.343	4.253 ± 0.365^a^	4.219 ± 0.081^a^	4.242 ± 0.288^a^	3.448 ± 0.211	4.189 ± 0.667	3.575 ± 0.596	5.034 ± 0.783^a^
C18:3	5.806 ± 0.178	7.345 ± 0.49^b^	5.696 ± 0.309	6.768 ± 0.358^a^	5.688 ± 0.614	8.376 ± 0.586^b^	6.059 ± 0.297	6.682 ± 1.092
C18:3n3	30.777 ± 0.465	31.353 ± 1.037	34.298 ± 2.817	32.517 ± 1.715	31.633 ± 2.849	32.311 ± 1.103	34.343 ± 1.283^a^	36.74 ± 0.781
C20:1	1.504 ± 0.033	2.054 ± 0.046^b^	1.555 ± 0.153	1.795 ± 0.095^b^	1.599 ± 0.204	2.133 ± 0.032^b^	1.946 ± 0.067^a^	1.977 ± 0.246^b^
C20:3	0.012 ± 0.003	0.089 ± 0.029^a^	0.055 ± 0.074	0.086 ± 0.049	0.026 ± 0.026	0.00	0.00	0.065 ± 0.067
C20:4	0.031 ± 0.001	0.06 ± 0.002^b^	0.042 ± 0.016	0.062 ± 0.004^b^	0.042 ± 0.017	0.005 ± 0.002^b^	0.007 ± 0.007^a^	0.047 ± 0.029
C22:2	0.00	0.081 ± 0.057	0.00	0.052 ± 0.003^b^	0.026 ± 0.037	0.011 ± 0.003^a^	0.008 ± 0.002^a^	0.08 ± 0.094
C24:0	0.085 ± 0.001	0.216 ± 0.014^b^	0.146 ± 0.067	0.242 ± 0.015^b^	0.141 ± 0.065	0.102 ± 0.004^b^	0.105 ± 0.012^a^	0.18 ± 0.067
C22:6	0.016 ± 0.004	0.093 ± 0.018^a^	0.045 ± 0.061	0.089 ± 0.006^b^	0.033 ± 0.045	0.014 ± 0.001	0.014 ± 0.002	0.074 ± 0.051
TFA	85.497 ± 1.581	94.788 ± 3.312^a^	97.377 ± 3.06^a^	99.878 ± 5.496^a^	87.56 ± 3.208	100.561 ± 4.856^b^	101.33 ± 4.842^b^	105.193 ± 3.618^b^

The table shows the average of all the observations from 4 repeated tests, in the form of mean ± standard error of representation, wild-type *C. reinhardtii* CC-849 represents a control, TFA represents the total fatty acid content. All data are expressed in mg/g (dry weight)

The data represent the means ± SD of three replicate experiments and were analyzed by Student’s t-test (n = 3)

^a^P <0.05; ^b^P <0.01. aRP1.1, aRP1.2, aRP1.3, aRP1.4 is transformed amicroRNA-PEPC1 into *C. reinhardtii*; aRP2.1, aRP2.2, aRP2.3 is transformed amicroRNA-PEPC2 into *C. reinhardtii*

**Table 2:** Various fatty acid compositions and contents of the transformants under conditions of heat shock for 30 min

Fatty acids	CC-849	aRP1.1	aRP1.2	aRP1.3	aRP1.4	aRP2.1	aRP2.2	aRP2.3
C12:0	0.049 ± 0.001	0.083 ± 0.002	0.083 ± 0.003	0.079 ± 0.001	0.085 ± 0.002	0.083 ± 0.003	0.085 ± 0.004	0.086 ± 0.001
C14:0	0.373 ± 0.028	0.467 ± 0.009	0.452 ± 0.004	0.44 ± 0.015	0.502 ± 0.007	0.416 ± 0.055	0.431 ± 0.009	0.435 ± 0.007
C16:0	17.646 ± 0.179	21.968 ± 0.931^b^	23.709 ± 1.406^b^	21.003 ± 0.758^b^	23.445 ± 0.293^b^	20.94 ± 0.719^b^	22.76 ± 0.406^b^	21.256 ± 1.432^b^
C16:1	2.666 ± 0.234	3.032 ± 0.145^a^	3.62 ± 0.072^b^	3.364 ± 0.038^b^	3.353 ± 0.044^b^	2.386 ± 0.293	3.092 ± 0.045^a^	2.789 ± 0.148
C18:0	3.37 ± 0.272	3.748 ± 0.114^a^	3.563 ± 0.023	3.126 ± 0.067	3.487 ± 0.02	3.51 ± 0.177	3.338 ± 0.036	3.059 ± 0.141
C16:4	20.756 ± 1.093	24.823 ± 0.314^b^	28.023 ± 1.839^b^	28.09 ± 0.848^b^	28.529 ± 0.146^b^	23.025 ± 0.781^a^	24.66 ± 1.453^b^	24.532 ± 0.254^b^
C18:1 t	2.393 ± 0.387	1.889 ± 0.246	2.625 ± 0.112	1.591 ± 0.06^b^	2.133 ± 0.183	1.958 ± 0.589	2.354 ± 0.05	2.011 ± 0.447
C18:2 t	9.163 ± 0.516	7.913 ± 0.555^a^	6.73 ± 0.123^b^	4.806 ± 0.261^b^	9.775 ± 0.354	7.605 ± 1.309	11.37 ± 0.673^b^	10.042 ± 1.465
C18:3	6.651 ± 0.565	7.272 ± 0.3	7.352 ± 0.123	7.06 ± 0.116	8.598 ± 0.273^b^	8.503 ± 0.591^b^	7.663 ± 0.365^a^	7.244 ± 0.289
C18:3n3	28.757 ± 1.697	33.663 ± 0.646^b^	36.882 ± 1.682^b^	35.235 ± 1.615^b^	39.886 ± 0.278^b^	31.335 ± 1.129^a^	35.924 ± 1.12^b^	35.765 ± 0.892^b^
C20:1	1.475 ± 0.023	1.483 ± 0.023	1.493 ± 0.038	1.656 ± 0.063^b^	1.663 ± 0.034^b^	1.516 ± 0.117	1.376 ± 0.096	1.354 ± 0.055^b^
C20:3	0.053 ± 0.009	0.093 ± 0.003^b^	0.096 ± 0.008^b^	0.095 ± 0.002^b^	0.093 ± 0.003^b^	0.098 ± 0.009^b^	0.097 ± 0.005^b^	0.095 ± 0.005^b^
C20:4	0.071 ± 0.002	0.105 ± 0.001^b^	0.107 ± 0.004^b^	0.106 ± 0.003^b^	0.119 ± 0.003^b^	0.111 ± 0.003^b^	0.118 ± 0.003^b^	0.119 ± 0.006^b^
C22:2	0.042 ± 0.003	0.081 ± 0.002^b^	0.084 ± 0.005^b^	0.079 ± 0.001^b^	0.083 ± 0.004^b^	0.098 ± 0.017^b^	0.085 ± 0.005^b^	0.082 ± 0.003^b^
C24:0	0.215 ± 0.003	0.381 ± 0.013^b^	0.382 ± 0.009^b^	0.339 ± 0.064^b^	0.375 ± 0.016^b^	0.36 ± 0.012^b^	0.363 ± 0.006^b^	0.299 ± 0.063^a^
C22:6	0.074 ± 0.005	0.145 ± 0.002^b^	0.158 ± 0.015^b^	0.142 ± 0.001^b^	0.142 ± 0.001^b^	0.143 ± 0.004^b^	0.142 ± 0.002^b^	0.144 ± 0.005^b^
TFA	93.754 ± 3.01	107.14 ± 3.306^b^	119.21 ± 6.55^b^	107.21 ± 3.7^b^	122.27 ± 0.86^b^	102.1 ± 1.15^b^	113.86 ± 3.41^b^	109.312 ± 4.99^b^

The data represent the means ± SD of three replicate experiments and were analyzed by Student’s t-test (n = 3)

^a^P <0.05; ^b^P <0.01. aRP1.1, aRP1.2, aRP1.3, aRP1.4 is transformed amicroRNA-PEPC1 into *C. reinhardtii*; aRP2.1, aRP2.2, aRP2.3 is transformed amicroRNA-PEPC2 into *C. reinhardtii*

**Table 3:** Various fatty acid compositions and contents of the transformants under heat shock conditions for 30 min ×3

Fatty acids	CC-849	aRP1.1	aRP1.2	aRP1.3	aRP1.4	aRP2.1	aRP2.2	aRP2.3
C12:0	0.046 ± 0.001	0.087 ± 0.001	0.074 ± 0.007	0.079 ± 0.002	0.08 ± 0.003	0.11 ± 0.031	0.084 ± 0.001	0.081 ± 0.001
C14:0	0.444 ± 0.016	0.625 ± 0.04	0.531 ± 0.047	0.556 ± 0.015	0.526 ± 0.011	0.69 ± 0.006	0.538 ± 0.007	0.523 ± 0.004
C16:0	20.208 ± 0.377	29.869 ± 0.812^b^	27.318 ± 2.346^b^	28.64 ± 0.535^b^	28.231 ± 0.61^b^	35.076 ± 1.781^b^	27.86 ± 0.999^b^	28.1 ± 0.558^b^
C16:1	3.865 ± 0.068	4.823 ± 0.826	4.98 ± 0.501^b^	4.68 ± 0.178^b^	4.114 ± 0.104^b^	4.335 ± 0.476	4.258 ± 0.128^b^	4.679 ± 0.108^b^
C18:0	3.257 ± 0.079	4 ± 0.177^b^	3.533 ± 0.156	4.014 ± 0.069^b^	3.84 ± 0.077^b^	5.094 ± 0.061^b^	3.27 ± 0.111	3.218 ± 0.054
C16:4	26.971 ± 0.282	36.832 ± 0.68^b^	34.999 ± 1.856^b^	38.697 ± 1.17^b^	35.366 ± 0.708^b^	33.142 ± 3.959^a^	36.463 ± 0.69^b^	38.57 ± 0.913^b^
C18:1 t	1.696 ± 0.177	4.201 ± 1.199^b^	2.764 ± 0.497^b^	2.22 ± 0.088^b^	1.872 ± 0.187	2.965 ± 0.32^b^	1.551 ± 0.119	1.857 ± 0.264
C18:2 t	5.687 ± 0.048	8.655 ± 1.497^b^	7.507 ± 0.558^b^	7.548 ± 0.165^b^	6.248 ± 0.065^b^	13.539 ± 0.682^b^	6.988 ± 0.43^b^	7.789 ± 0.208^b^
C18:3	8.042 ± 0.054	9.797 ± 0.304^b^	8.918 ± 0.353^b^	9.98 ± 0.229^b^	9.17 ± 0.472^b^	13.672 ± 1.253^b^	9.549 ± 0.318^b^	10.004 ± 0.31^b^
C18:3n3	34.281 ± 0.647	48.215 ± 2.613^b^	44.218 ± 2.514^b^	48.18 ± 1.527^b^	45.387 ± 1.151^b^	46.848 ± 5.121^a^	45.256 ± 0.94^b^	47.64 ± 1.178^b^
C20:1	1.793 ± 0.051	2.179 ± 0.348	1.751 ± 0.079	2.154 ± 0.05^b^	2.165 ± 0.089^b^	2.077 ± 0.276	2.099 ± 0.041^b^	2.161 ± 0.086^b^
C20:3	0.048 ± 0.001	0.097 ± 0.003^b^	0.089 ± 0.01^b^	0.093 ± 0.001^b^	0.097 ± 0.005^b^	0.125 ± 0.05^a^	0.111 ± 0.034^a^	0.095 ± 0.005^b^
C20:4	0.063 ± 0.001	0.124 ± 0.019^b^	0.104 ± 0.006^b^	0.11 ± 0.004^b^	0.107 ± 0.001^b^	0.145 ± 0.046^b^	0.105 ± 0.002^b^	0.105 ± 0.003^b^
C22:2	0.041 ± 0.002	0.091 ± 0.009^b^	0.078 ± 0.009^b^	0.081 ± 0.002^b^	0.083 ± 0.002^b^	0.111 ± 0.046^b^	0.084 ± 0.002^b^	0.081 ± 0.003^b^
C24:0	0.266 ± 0.012	0.455 ± 0.006^b^	0.4 ± 0.027^b^	0.415 ± 0.012^b^	0.415 ± 0.005^b^	0.532 ± 0.11^b^	0.389 ± 0.009^b^	0.386 ± 0.002^b^
C22:6	0.073 ± 0.004	0.14 ± 0.001^b^	0.142 ± 0.023^b^	0.144 ± 0.003^b^	0.144 ± 0.003^b^	0.188 ± 0.085^a^	0.142 ± 0.002^b^	0.147 ± 0.004^b^
TFA	106.78 ± 12.24	150.19 ± 2.882^b^	137.406 ± 8.66^b^	140.212 ± 9.72^b^	137.845 ± 4.65^b^	158.65 ± 18.42^b^	138.743 ± 8.4^b^	145.44 ± 11.61^b^

For applying heat shock (HS), 400 mL cells (WT and transgenic algae) in mid-logarithmic phase were incubated at 40 °C and light intensity 20 μmol/m^2^/s for 30 min. As for triple heat shocks, a 4-h recovery period was utilized between each heat shock

The data represent the means ± SD of three replicate experiments and were analyzed by Student’s t-test (n = 3)

^a^P <0.05; ^b^P <0.01. aRP1.1, aRP1.2, aRP1.3, aRP1.4 is transformed amicroRNA-PEPC1 into *C. reinhardtii*; aRP2.1, aRP2.2, aRP2.3 is transformed amicroRNA-PEPC2 into *C. reinhardtii*

**Fig. 4:**
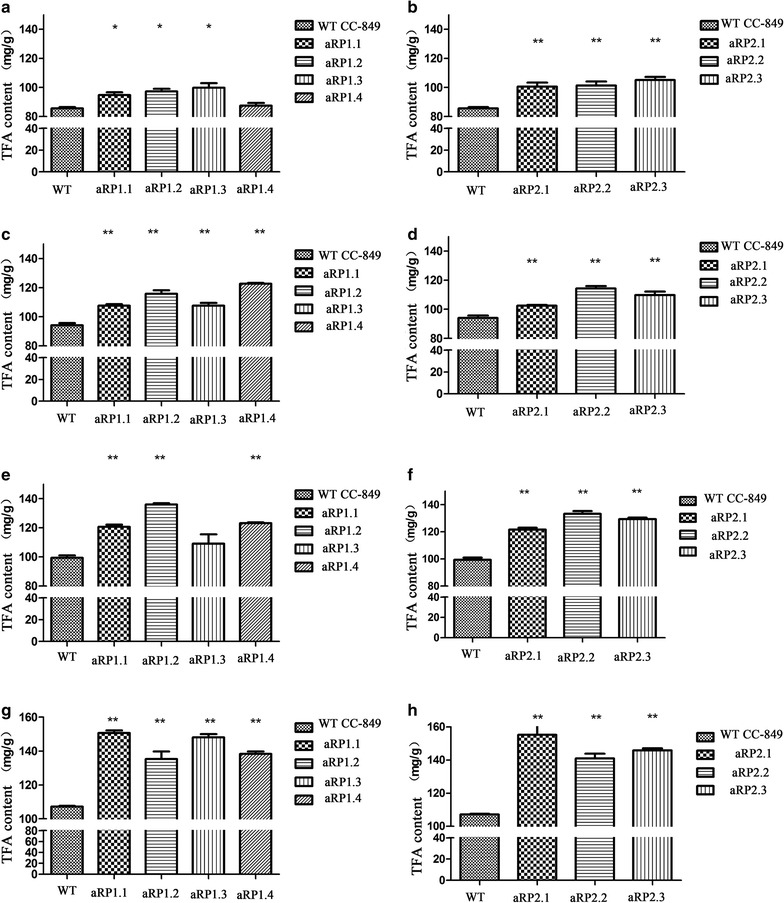
Total fatty acid content in transformants: **a** aRP1 before heat shock; **b** aRP2 before heat shock; **c** aRP1 after ×1 heat shock; **d** aRP2 after ×1 heat shock; **e** aRP1 after ×2 heat shock; **f** aRP2 after ×2 heat shock; **g** aRP1 after ×3 heat shock; and **h** aRP2 after ×3 heat shock

After HS treatments for three times, the TFA of WT was increased by 24.9% in the control. The mutants had significantly higher TFA production than their respective control before heat shock with 58.3% (aRP1.1), 41% (aRP1.2), 47.6% (aRP1.3), 57.3% (aRP1.14), 57.8% (aRP2.1), 36.9% (aRP2.2), and 38.3% (aRP2.3) (Table 3). What is more, compared with WT cells that underwent HS treatments three times, transgenic algal cells had significantly higher TFA than the respective WT controls, with increases of 40.7% (aRP1.1), 28.7% (aRP1.2), 38.2% (aRP1.3), 29.1% (aRP1.14), 48.6% (aRP2.1), 29.9% (aRP2.2), and 36.2% (aRP2.3), respectively (Fig. 4). The results showed that the heat shock-induced overexpression of *CrPEPCs* amiRNAs significantly increases the TFA content of transgenic *Chlamydomonas* strains.

The increase in TFA content of transgenic algae was contributed by increases of nearly all the different fatty acids (Table 3). Interestingly, C18:3n3, C16:0, and C16:4 were among the most significant constituents after heat shocks. The fatty acid C18:3n3 had the highest content which increased from 9.93 to 13.9 mg/g (DW) with an average increase of 35.75% in transgenic strains when comparing with 3 × HS WT; the C16:0 contents increased from 7.11 to the maximum 16.87 mg/g (DW); C16:4 content increased from 6.17 to 11.6 mg/g (DW), with a 43% increase in the transformant aRP2.3. Moreover, although the low contents of long-chain fatty acids such as C20:3, C22:2, and C22:6 were observed, they significantly increased from 85 to 160% according to GC–MS after 3 × HS (Table 3). These results suggest that HS treatment to induce the expression of amiRNAs can effectively down-regulate *CrPEPC* genes’ expression in *C. reinhardtii*, resulting in an increase of fatty acid synthesis with the most significant increase occurring for C16 to C22 fatty acids.

## Discussion

In this study, the WMD3 platform was employed to design amiRNAs against the *Chlamydomonas reinhardtii CrPEPC1* and *CrPEPC2* genes, and the amiRNAs were successfully induced by the heat shock protein (*Hsp70A*) promoter-driven expression system. Using 42 °C as the hHS, we observed significantly increased amiRNAs, *aRP1* and *aRP2* against *CrPEPC1* and *CrPEPC2*, respectively, which were overexpressed at least 16 times after HS. *Hsp70A* promoter known as heat-inducible promoter was described a lot, showing that the transcription levels of foreign genes controlled by *Hsp70A* promoter were significantly increased after incubation at 42 °C for 30 min [22, 23]. The similar results were observed here that amiRNA expression reached the highest level after incubation at 42 °C for 30 min. We used multi-heat shocks to maximize amiRNAs’ expression. In this research, the expression level of amiRNAs was higher than the others [24, 25]. Till now, there was no report on the increase of TFA by amiRNA technology in microalgae. Our results showed that the expression of amiRNAs was increased about 28 times after HS, while that of *CrPEPCs* were decreased 90%, leading to a 48.6% increase in TFA. Here, we showed a highly effective miRNA expression system that was inducible and significantly increased TFA in *C. reinhardtii*.

It is well known that there are two PEPC isoenzymes in *C. reinhardtii*, *CrPEPC1* and *CrPEPC2* [21]. *CrPEPC1* is proposed as a structurally homologous tetramer with maximum catalytic activity, while *CrPEPC2* works as a multi-subunit complex whose expression pattern is more complex than *CrPEPC1* [26, 27]. Activities of *CrPEPC1* were decreased by 39–50% using RNAi technology [12, 28]. Here we observe the differential transcription regulation between *CrPEPC1* and *CrPEPC2* under HS. For example, after HS, a sharp rise of *CrPEPC1* in the WT algal cells was observed. On the other hand, the gene expression of *CrPEPC2* declined constantly after HS. Results from several labs proved that it was reliable to silence target gene using amiRNA in *C. reinhardtii* [19, 24, 25]. It was shown that the amiRNAs designed in our experiment showed high specificity and efficiency compared to *CrPEPCs* . After HS, the expression levels of aRP1 and aRP2 were increased 19–28 times, respectively, resulting in specific silencing of *CrPEPCs*. Although *CrPEPC1* and *CrPEPC2* had different response patterns to HS treatment, their effective down-regulation by amiRNAs under HS treatment compared with the WT was similar to significant reductions. For instance, aRP1 reduced the expression level of *CrPEPC1* by ~93% compared to that in WT, and aRP2 down-regulated *CrPEPC2* expression levels by up to 90% compared to WT. Furthermore, we did not detect any off-target phenomenon in all transgenic algae even after 1-year cultivation in the lab, indicating the stability of transgenic algae. Additionally, the inducible expression system for miRNA can reduce the cell damage caused by the expression of amiRNA. Therefore, our results showed that we succeeded in obtaining amiRNAs that were highly specific to silencing of *CrPEPCs*.

The observations demonstrate that we have constructed amiRNAs to inhibit the expression of *CrPEPC* genes in *Chlamydomonas* successfully. The designed amiRNAs own a high degree of complementarity with *CrPEPC* mRNAs to ensure that the RNA-induced silencing complex could bind to *CrPEPC* mRNAs resulting in the cleavage of them. It appears that decreasing *CrPEPC* mRNA abundance led to efficiently silencing their expression, redirecting carbon flux to fatty acid synthesis. As a result, the down-regulated *CrPEPCs*’ expression levels decreased the protein biosynthesis, and thus more carbon source flowed to the fatty acid synthesis pathway, resulting in the TFA increase. Although it was previously claimed that the activity of *CrPEPC1* was inhibited by RNA interference, TAG levels only increased 20% as observed in a previous study [28], which never met the demand of industrial production. Moreover, using RNA interference technology requires a multi-step vector strategy with off-target effects so that it is likely not the best strategy for the manipulation of fatty acid synthesis [17]. Hence, the amiRNA with high specificity and efficiency was needed to improve TFA production in microalgae. In the present study, positive results were observed in the case of *CrPEPC1* and *CrPEPC2*, the down-regulation of which resulted in significantly improved TFA content of microalgae, with a highest increase of 48.6% [158.65 mg/g (DW)] compared to that of WT.

We conducted GC–MS analyses to define the specifically changed FA components and found a significantly increase in long-chain fatty acid contents using our amiRNA method. Especially, the contents of C16 to C18 fatty acids generally increased by more than 30%. This is consistent with a previous report in oilseed rape seeds, with a substantial increase of C18 fatty acids following a sharp reduction of PEPC activity [29]. Moreover, a notable increase in the contents of long-chain fatty acids such as C20:3, C22:2, and C22:6 was also observed, which is useful for producing biodiesel.

## Conclusions

The finding of this study indicated that overexpressing amiRNAs with an *Hsp70A* promoter remarkably inhibited the transcription of *CrPEPCs*, resulting in a significant increase of TFA in *Chlamydomonas*. This is the first report in which amiRNA was employed to increase TFA in *Chlamydomonas*. Although the expression levels of *CrPEPC1* and *CrPEPC2* are regulated in different ways, the amiRNAs remarkably inhibited them both at the transcription level, which made it possible to silence *CrPEPC1* and *CrPEPC2* at the same time. Moreover, TFA in transgenic algae was further increased with the help of multi-heat shocks and the contents of C16 and C22 fatty acids were significantly increased. In summary, this study demonstrates a high potential for the use of efficient *CrPEPC* amicroRNAs to improve the TFA in *Chlamydomonas* and provides a new possible way to increase the TFA production in other microalgae such as diatom for biodiesel.

## Methods

### Algal strains and culture conditions

A cell wall-deficient *C. reinhardtii* strain, CC-849, was obtained from the *Chlamydomonas* Genetic Center of Duke University (Duke University, Durham, NC, USA). Cells were cultured in TAP (Tris-acetate-phosphate) medium at 25 °C and under continuous cool-white fluorescent lamps (~20 μmol photons/m^2^ s) (normal condition, NT). For HS induction, 400 mL cells (WT and transgenic) in mid-logarithmic phase were incubated in a water bath at 42 °C for 30 min. After the treatment, algal cells were subjected to DNA/RNA extraction and GC–MS detection.

### amiRNA constructs

The Web MicroRNA Designer (WMD3) website (http://wmd3.weigelworld.org/cgi-bin/webapp.cgi) was used to design mature amicroRNA-*PEPC1*/2 to target the *CrPEPC1*/2 genes [17]. ami-PEPC1 (5´-TATTGGATTGAAAGGTCGCTA-3´) and ami-PEPC2 (5´-TTAACCAAACATTTTCGGCAC-3´) are complementary to nucleotides 117–137 bp of the coding region of *CrPEPC1* and *CrPEPC2*, respectively. Then ami-PEPC1 and ami-PEPC2 replaced the mature sequence of *C. reinhardtii* endogenous miRNA-cre-MIR1162 (Accession Number: MI0006223) [24], to obtain amicroRNA-PEPC1 (aRP1) and amicroRNA-PEPC2 (aRP2), respectively (see Additional file 3). The constructed amicroRNA-PEPC1/2 (154 bp) was commercially synthesized in vitro and was cloned in plasmid pUC57 (Sangon Biotech Co., Ltd, Shanghai, China).

**Fig. 5:**
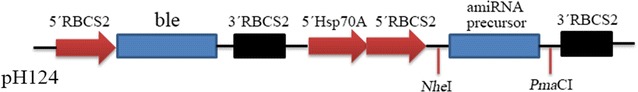
Schematic diagram of the construct pH-amicroRNA-PEPC1/2. pH124 contained the ampicillin- and zeomycin-resistant genes and a strong heat-inducible promoter (*Hsp70A*-*RBCS2*). The DNA fragments of amicroRNA-PEPC1 (aRP1) and amicroRNA-PEPC2 (aRP2) were ligated to the restriction enzyme sites, *Nhe*I and *PmaC*I, within the *Chlamydomonas* transformation vector pH124, resulting in pH-amicroRNA-PEPC1 (pH-aRP1) and pH-amicroRNA-PEPC2 (pH-aRP2), respectively. The amiRNA precursors included amicroRNA-PEPC1/2, which were overexpressed under *Hsp70A* promoter

The expression vector pH124 containing the Ampicillin- and Zeomycin-resistant genes and a strong heat-inducible promoter (*Hsp70A*-*RBCS2*) was constructed in our lab [30]. Enzymatic digestion sites of *Nhe*I (GCTAGC) and *Pma*C I (CACGTG) were added at 5′- and 3′-end of aRP1/2, respectively. The DNA fragments of amicroRNA-PEPC1 (aRP1) and amicroRNA-PEPC2 (aRP2) were then ligated to the restriction enzyme sites, *Nhe*I and *PmaC*I, in the *Chlamydomonas* transformation vector pH124, resulting in pH-amicroRNA-PEPC1 (pH-aRP1) and pH-amicroRNA-PEPC2 (pH-aRP2), respectively (Fig. 5).

### DNA-PCR analysis of transgenic alga

PCR analysis was utilized to confirm the successful transformation of our target amicroRNA-PEPC1/2. Genomic DNA was extracted from both WT and transgenic algae using the DNeasy kit (Takara, Japan). PCR was carried out according to the standard protocols to verify the presence of amicroRNA-PEPC1/2 on transgenic alga. PCR was performed using the primer pair pH124-F (5′TGACCTCCACTTTCAGCGACA3′) and pH124-R (5′ACTTGAGAGCAGTATCTTCCATCCA3′), which resulted in an amplicon of about 675 bp. PCR conditions were as follows: incubation at 95 °C for 2 min, followed by 25 cycles of 95 °C for 30 s, 60 °C for 15 s, and 72 °C for 15 s, plus a final extension for 7 min. All the amplified products were purified and verified by sequencing analyses (Sangon Biotech., Shanghai, China).

### RNA extraction and RT-PCR

Total RNA was extracted using RNAiso Plus for Total RNA kit and DNA synthesis performed using PrimeScript™ Double-Strand cDNA Synthesis Kit (Takara Biotechnology Co., LTD, Dalian, China) according to the manufacturer’s instructions. The miRNA was extracted using RNAiso for Small RNA (Takara Biotechnology Co., LTD, Dalian, China), and reversed and polyadenylated using SYBR^®^ PrimeScript™ miRNA RT-PCR Kit (Takara Biotechnology Co., LTD, Dalian, China) according to the manufacturer’s instructions. The amplified positive products were verified by sequencing analyses (Sangon Biotech., Shanghai, China).

### Quantitative real-time PCR (qRT-PCR)

To quantitatively detect changes of amicroRNA-PEPC1/2 and *CrPEPC1*/2 expression in both wild-type and transgenic algae, qRT-PCR was performed with Applied Biosystems 7300 real-time PCR System (Framingham, MA, USA), and the primers used are listed in Additional file 4: Table S1. The standard protocol was applied to *CrPEPC1*/2 expression detection using SYBR Premix Ex Taq™II (Takara, Japan) according to the manufacturer’s instruction, while the expression of amicroRNA-PEPC1/2 was detected as described previously [31]. PCR conditions were as follows: one step of 95 °C for 30 s, followed by 40 cycles of 95 °C for 5 s and 60 °C for 30 s. Analysis of the melting curve of amplicons was used to test the specificity of the primers. The β-actin gene and U4 snoRNA were used as the reference genes in qRT-PCR detection of *CrPEPCs* and amicroRNA-PEPC1/2, respectively. Data with an *R* ^2^ above 0.998 were analyzed using the 2^−ΔΔCt^ program [32]. Three technical replicates and two biological replicates were accustomed.

### Nuclear transformation of *C. reinhardtii*

Genetic transformation of *C. reinhardtii* CC-849 was carried out using the “glass beads” method, according to Kindle [33] and Wang et al. [34].

### Fatty acid methyl ester (FAME) transformation and FAME analysis

Total lipid extraction was performed as described by Lu et al. [35] with slight modifications. Briefly, 20 mg of lyophilized cells were suspended in 1 mL of 2 M NaOHCH_3_OH solution, shaken (100 rpm) for 1 h at 25 °C, and incubated at 75 °C for 15 min. After cooling down, the mixture was spiked with 1 mL of 4 M HCl–CH_3_OH and pH was set to below 2.0 with HCl, followed by incubation at 75 °C for 15 min. After that, FAMEs were extracted with 1 mL hexane, shaken by hand for 30 s, and then centrifuged at 4000*g* for 2 min. The hexane phase was collected and stored at −20 °C for further GC–MS analysis. 100 μg of C19 FA was added before extraction to estimate the recovery rate. Qualification and quantification of FAMEs were performed on a Thermo Trace GC Ultra Gas Chromatograph coupled to Thermo PolarisQ Mass Spectrometer which was equipped with an HP-5MS column (30 m × 0.25-mm id, film thickness 0.25 μm). The temperature of the injector was maintained at 250 °C. Helium was used as the carrier gas and ions were generated by a 70 eV electron beam and the mass range scanned was 50–650 *m*/*z* at a rate of 2 scan/s. The oven temperature for FAME analysis was initially maintained at 70 °C for 5 min followed by a temperature rate of 5 °C/min to 200 °C and then held for 5 min, 5 °C/min to 204 °C and then held for 2 min, 5 °C/min to 220 °C and then held for 3 min, and 5 °C/min to 255 °C and then held for 5 min. Peak identification was performed by matching the mass spectra of each compound with the National Institute of Standards and Technology mass spectral library. Automatic peak deconvolution was processed with MassLynx software (V4.1, Waters Corp., USA) [32]. The datasets of FAME profiling for further analysis were obtained by normalizing with the internal standards in the same chromatograms, respectively.

### Statistical analysis

All exposure experiments were repeated three times independently, and data were reported as the mean with standard deviation (SD). For gene expression experiments, quantitative real-time PCR analysis was performed using the BioRAD iQ5 software. For each gene, the fold change expressed as mean ± SD (% control) was calculated using the (standard curve) approximation corrected for primer efficiency and normalized to housekeeping gene β-actin expression values. Statistical analyses were performed using the Student’s t test and Pearson Correlation analysis (SPSS13.0). For all of the data analysis, a *p* value <0.05 was considered statistically significant.

## Additional files

**Additional file 1: Figure S1.** PCR verification of the amiRNAs from transgenic algae. M: DL 2000 marker; 1: positive control; 2–4: transgenic algae with amicroRNA-PEPC1, 5–7: transgenic algae with amicroRNA-PEPC2.

**Additional file 2: Figure S2.** The growth curve of transgenic *C. reinhardtii*. aRP1.1, aRP1.2 were derived from individual amicroRNA- PEPC1 transformants, and aRP2.1, aRP2.2, aRP2.3 were from amicroRNA- PEPC2 transformants. *C. reinhardtii* CC-849 is the wild type. Introduction of amicroRNA- PEPC1 and amicroRNA- PEPC2 has no effect on the growth of transgenic algae.

**Additional file 3.** Sequence of amicroPEPCs inserted into *C. reinhardtii* endogenous miRNA- cre- MIR1162.

**Addition file 4: Table S1.** Primers used in the quantitative Real-Time PCR.

## Abbreviations

PEP: phosphoenolpyruvate
PEPC: phosphoenolpyruvate carboxylase
PK: pyruvate kinase
OAA: oxaloacetic acid
TCA: tricarboxylic acid cycle
FAS: fatty acid synthase
ACCase: acetyl coenzyme A carboxylase
PDC: pyruvate decarboxylase
MACT: malonyl-CoA:ACP transacylase
amiRNA: artificial microRNA
ACCase: acetyl coenzyme A carboxylase
TFA: total fatty acid
HS: heat shock
NT: normal condition
DW: dry weight
