# Supplementary figures and images for: Artificial miRNA inhibition of phosphoenolpyruvate carboxylase increases fatty acid production in a green microalga Chlamydomonas reinhardtii
Source: Biotechnol Biofuels. 2017 Apr 13;10:91. doi: 10.1186/s13068-017-0779-z (PMC5390379; doi:10.1186/s13068-017-0779-z)

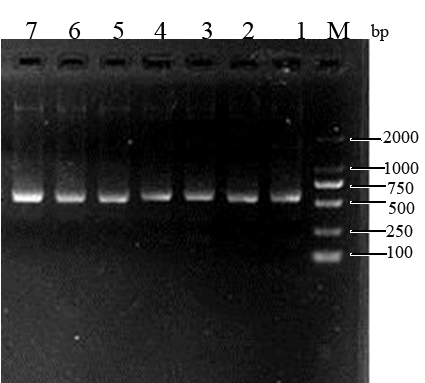

Supplement: Supplementary file 1 — Additional file 1: Figure S1. PCR verification of the amiRNAs from transgenic algae. M: DL 2000 marker; 1: positive control; 2–4: transgenic algae with amicroRNA-PEPC1, 5–7: transgenic algae with amicroRNA-PEPC2. [file 13068_2017_779_MOESM1_ESM.tif]

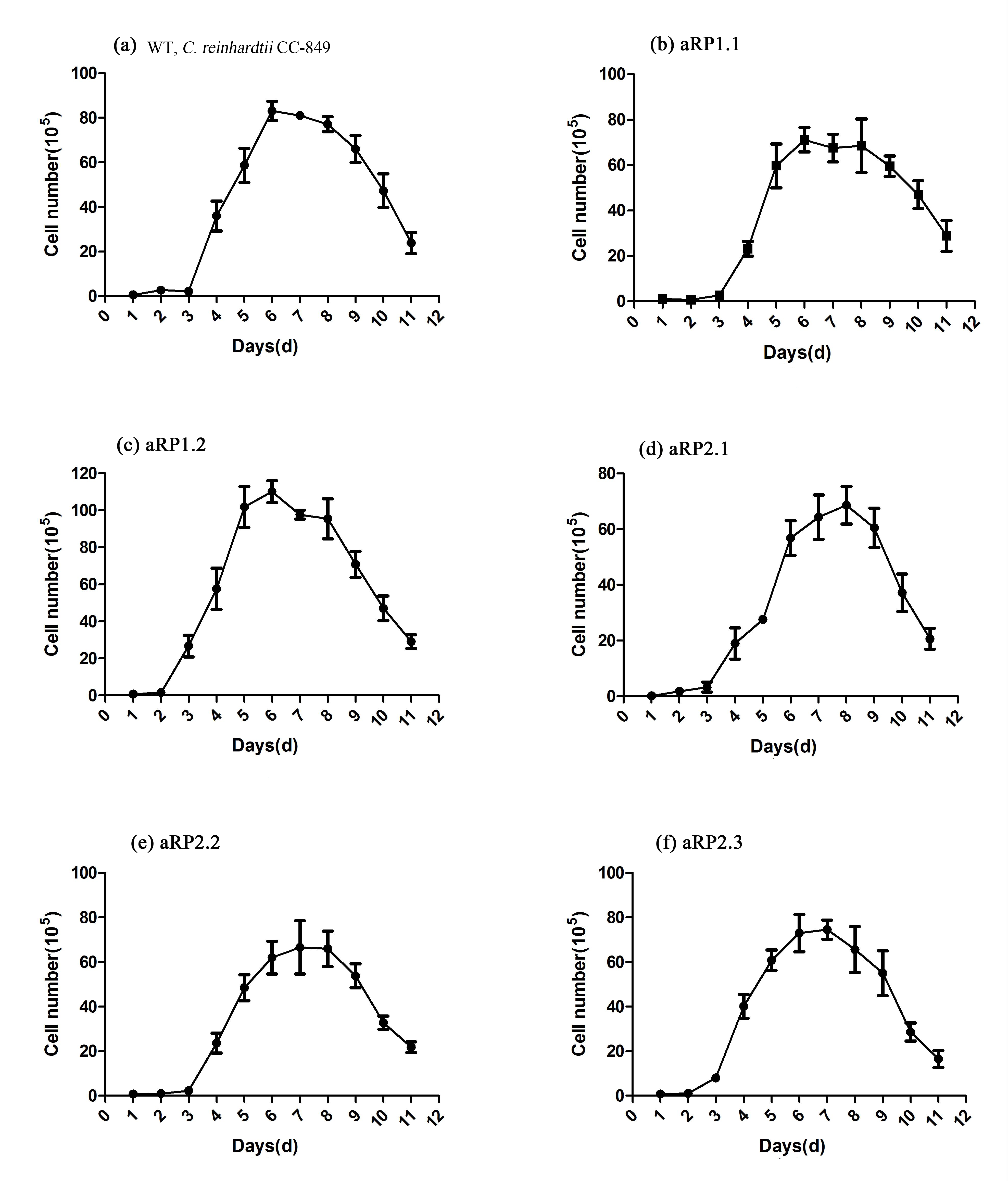

Supplement: Supplementary file 2 — Additional file 2: Figure S2. The growth curve of transgenic C. reinhardtii. aRP1.1, aRP1.2 were derived from individual amicroRNA- PEPC1 transformants, and aRP2.1, aRP2.2, aRP2.3 were from amicroRNA- PEPC2 transformants. C. reinhardtii CC-849 is the wild type. Introduction of amicroRNA- PEPC1 and amicroRNA- PEPC2 has no effect on the growth of transgenic algae. [file 13068_2017_779_MOESM2_ESM.tif]
