# Supplementary material for: Artificial miRNA inhibition of phosphoenolpyruvate carboxylase increases fatty acid production in a green microalga Chlamydomonas reinhardtii
Source: Biotechnol Biofuels. 2017 Apr 13;10:91. doi: 10.1186/s13068-017-0779-z (PMC5390379; doi:10.1186/s13068-017-0779-z)
Supplement: Supplementary file 3 — Additional file 3. Sequence of amicroPEPCs inserted into C. reinhardtii endogenous miRNA- cre- MIR1162. [file 13068_2017_779_MOESM3_ESM.docx]

Sequence of amicroPEPCs inserted into *C. reinhardtii* endogenous miRNA- cre- MIR1162

>amicroRNAs-cre-MIRpepc1

GCTAGCGCGGGGCCCUGACACCACUGCGGCCGC**uagcgaccaaacaauccaaua**CCGCGCCUGGACCCGAGGGAGGACCCCUCGGGACCCGGUACGUCG**UAUUGGAUUGAAAGGUCGCUA**GGUCGCGGUGGGGUCAGGUCCUUCCGCCACGTG

>amicroRNAs-cre-MIRpepc2

GCTAGCGCGGGGCCCUGACACCACUGCGGCCGC**gugccgaaaauguuugguuaa**CCGCGCCUGGACCCGAGGGAGGACCCCUCGGGACCCGGUACGUCG**UUAACCAAACAUUUUCGGCAC**GGUCGCGGUGGGGUCAGGUCCUUCCGCCACGTG
