# Supplementary material for: Artificial miRNA inhibition of phosphoenolpyruvate carboxylase increases fatty acid production in a green microalga Chlamydomonas reinhardtii
Source: Biotechnol Biofuels. 2017 Apr 13;10:91. doi: 10.1186/s13068-017-0779-z (PMC5390379; doi:10.1186/s13068-017-0779-z)
Supplement: Supplementary file 4 — Addition file 4: Table S1. Primers used in the quantitative Real-Time PCR. [file 13068_2017_779_MOESM4_ESM.docx]

Table S1. Primers used in the quantitative Real-Time PCR

| Primer name | Sequence (5´ to 3´) | Target gene |
| --- | --- | --- |
| PEPC1-F | ACTTGCTGTGTCCAAATGCT | CrPEPC1 |
| PEPC1-R | CAAGCGTTCCATCCAAATCC |  |
| PEPC2-F | CGCTGCTCATCTCCATCAA | CrPEPC2 |
| PEPC2-R | TCCATCTCCACCTCCACCTC |  |
| actin-F | ACCCCGTGCTGCTGACTG | β-actin |
| actin-R | ACGTTGAAGGTCTCGAACA |  |
| U4-F | CAAAAGGCCCGACAGAAAT | U4 snoRNA |
| U4-R | GTGAGGTCTAACCGAGTCGC |  |
| amiRNAP1-F | TATTGGATTGAAAGGTCGCTA | amiRNA-PEPC1 |
| amiRNAP2-F | TTAACCAAACATTTTCGGCAC | amiRNA-PEPC2 |
